# Light-Driven Photobiocatalytic Oxyfunctionalization in a Continuous Reactor System without External Oxygen Supply

**DOI:** 10.1021/acssuschemeng.4c08560

**Published:** 2025-03-05

**Authors:** Lenny Malihan-Yap, Qian Liang, Alessia Valotta, Véronique Alphand, Heidrun Gruber-Woelfler, Robert Kourist

**Affiliations:** † Institute of Molecular Biotechnology, Graz University of Technology, Petersgasse 14, 8010 Graz, Austria; ‡ Institute of Process and Particle Engineering, Graz University of Technology, Inffeldgasse 13, 8010 Graz, Austria; § CNRS, Cent Marseille, iSm2, Aix Marseille Univ, F-13397 Marseille, France; ∥ ACIB GmbH, Krengasse 37, 8010 Graz, Austria

**Keywords:** cyanobacteria, Baeyer−Villiger oxidation, monooxygenase, flow reactor, light-driven biotransformations, oxygenic photosynthesis, *Synechocystis*

## Abstract

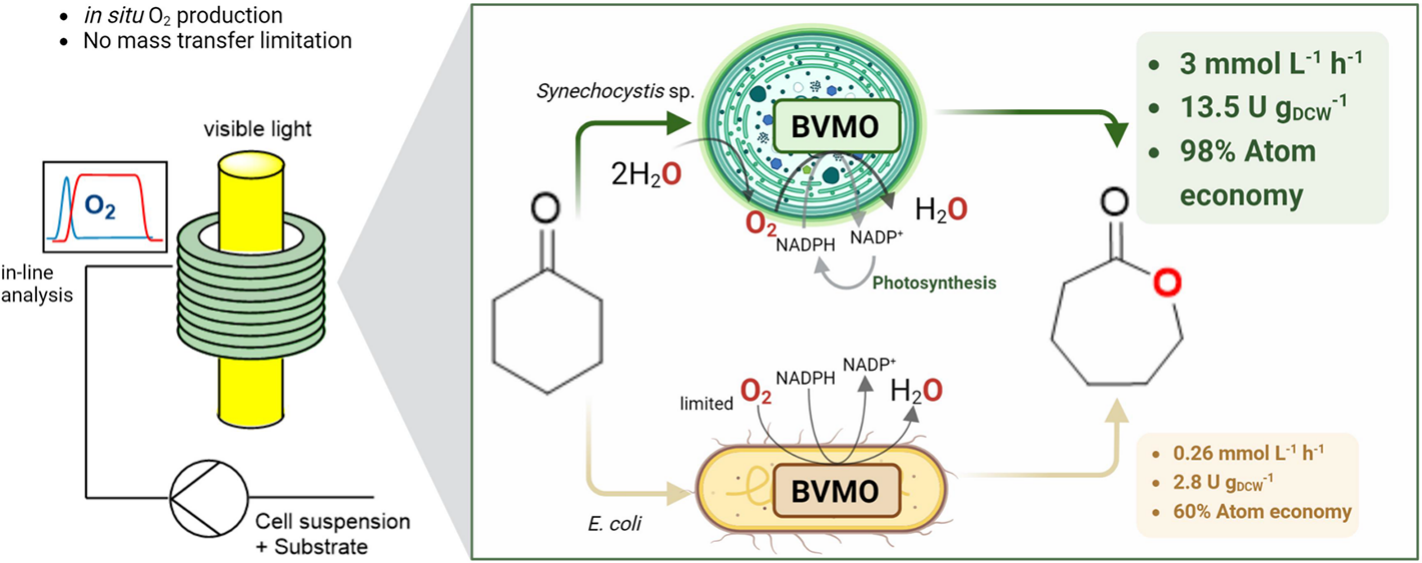

Oxygenases catalyze C–H oxyfunctionalization under mild reaction conditions and often display outstanding selectivity. However, their utilization is hampered by the difficulty of transporting oxygen across the gas–liquid interface, which is particularly problematic for continuous reactor systems and can only be alleviated by high pressure or the use of complex oxygen-permeable materials. Herein, oxygen is directly released into the medium by the phototrophic cyanobacterium *Synechocystis* sp. PCC 6803 expressing the genes of a Baeyer–Villiger Monooxygenase from *Burkholderia xenovorans* to drive the oxidation of cyclohexanone for the production of the polymer precursor, ε-caprolactone. The rates at which photosynthetic oxygen can solely drive the oxidation were determined by performing the reaction in a continuous coil reactor with a very limited external oxygen supply. In heterotrophic nonoxygen-producing **Escherichia coli** expressing the same gene, a 10-fold lower specific activity was observed when the oxidation was performed in the coil reactor compared with batch mode underlining the impact of oxygen-limitation on the volumetric productivity. In contrast, cyanobacterial whole cells showed activities of 16.7 and 13.5 U g_DCW_^–1^ in nonoxygen-limited batch and oxygen-limited continuous flow, respectively. Net oxygen production of the whole-cell biocatalyst during the reaction led to a steady-state oxygen concentration allowing volumetric productivities as high as 3 mmol L^–1^ h^–1^ highlighting the advantages of photoautotrophic production systems for oxyfunctionalization under oxygen-limiting conditions. Moreover, the space-time yield of the reaction was improved 7-fold (2.8 vs 0.4 g L^–1^ h^–1^) by utilizing the continuous coil reactor compared to the batch mode. The combination of flow catalysis and photosynthetic oxygen production can overcome current limitations in photo(bio)oxidation and achieve significant improvements in terms of volumetric productivity enabling more sustainable chemical synthesis. This approach using whole-cells of cyanobacteria achieves a notably lower ratio of waste to product (E-factor) and higher atom economy compared with oxidation mediated by **Escherichia coli**.

## Abstract 2

Photosynthetic oxygen was utilized to solely drive an enzymatic oxidation in recombinant cyanobacteria harboring a Baeyer−Villiger Monooxygenase for the production of a polymer precursor in an oxygen-limited illuminated flow reactor.

## 1 Introduction

Continuous flow chemistry, wherein chemical processes take place in a continuously flowing stream, has emerged as a significant technology in recent years. Due to the high level of control over process parameters, reactions that are otherwise not feasible in batch reactors can be performed.^1−3^ In addition, enhancement of reaction rates was shown in flow due to mass and heat transfer intensification owing to the miniaturization of reactor dimensions.^4,5^ Among the many recent examples of reactions that exploited the advantages of flow chemistry, the synthesis of active pharmaceutical ingredients,^6−9^ natural products,^10^ nucleosides^11,12^ and screening of Suzuki reactions stand out.^13^ Flow reactors are also systems of choice for photocatalytic reactions^14,15^ and chemoenzymatic cascades.^16−18^

Oxidative transformations are one of the most important reactions for the synthesis of pharmaceutical intermediates and fine chemicals.^19^ Traditional methods employing chemical oxidants are often energy-intensive, display poor selectivity, and generate undesirable byproducts. In addition, thermal control of batch rectors in highly oxidizing systems is difficult especially when utilizing pure O_2_ in the presence of other flammable solvents.^20^ In particular, Baeyer–Villiger oxidation uses organic peracids which are hazardous and pose ecological safety issues.^21,22^

Biocatalysis offers several advantages that may alleviate these drawbacks due to the high selectivity of many enzymes and near-ambient process requirements resulting in reduced byproduct formation and energy savings.^23^ As an example, whole cells of **Escherichia coli** expressing the genes of several flavin-dependent monooxygenases were investigated for the production of the platform chemical, ε-caprolactone^22,24^ and for the oxidation of bicyclo[3.2.0]hept-2-en-6-one.^25−27^ Albeit being sustainable, oxidation reactions involving enzymes in aqueous media suffer from the poor solubility of O_2_, limiting reaction rates.^28^ In addition, chemoheterotrophic *E. coli* respire O_2_ at a rate of at least *ca.* 60 μmol min^–1^ g_DCW_^–1^ competing with the reaction and thereby decreasing product formation rates.^29,30^ Furthermore, the dependence of the majority of oxygenases on nicotinamide cofactors [NAD(P)H] poses another challenge for the development of a cheap and efficient cofactor recycling system for it to be industrially feasible.^22,29^ Another hurdle in utilizing heterotrophic *E. coli* is the addition of sacrificial cosubstrates in stoichiometric amounts such as glucose or glycerol to fuel their metabolism and to serve as electron sources decreasing the atom economy and concurrently increasing the environmental factor (E-factor).^31,32^

Homogeneous oxygen produced by cyanobacteria directly in the medium could be a viable option to overcome the gas–liquid mass transfer limitation for oxygen-dependent whole-cell biocatalysis. This concept was first established by Hoschek et al.^33^ using an alkane monooxygenase in the cyanobacterium *Synechocystis* sp. PCC 6803 (hereafter *Synechocystis*). However, as they used a very slow biocatalyst with a specific activity of only 0.9 U g_DCW_^–1^, it remains unclear which volumetric productivity can be sustained solely by oxygenic photosynthesis in systems with limited external oxygen supply. In batch mode, *in situ* O_2_ production was also exploited to drive other oxidation reactions using Baeyer–Villiger Monooxygenases (BVMOs)^34−37^ and cytochrome P450 monooxygenases (CYP450).^38−41^ Furthermore, the cyanobacterial pool of reducing equivalents in the form of β-nicotinamide adenine dinucleotide phosphate (NADPH) regenerated in the cell has already been shown to catalyse and sustain various NADPH-dependent reduction reactions^42−49^ initially shown by us using the asymmetric reduction of C=C.^32^

Despite these advantages, the industrial application of light-driven oxidation using cyanobacteria has been hampered by the availability of light.^14,49^ This was demonstrated when a BVMO reaction was translated from small scale (*V* = 1 mL) to a 2 L stirred tank reactor resulting in a 2-fold decrease in activity (from 60 to 30 U g_DCW_^–1^).^37^ In batch reactions, increasing the path length decreases the light penetration exponentially following the Bouguer-Lambert–Beer law.^50^ Hence, novel reactor concepts have been formulated to decrease the light path length including internal illumination,^45^ glass capillary reactors,^51^ and recently a continuous coil reactor in flow.^49^ In the latter, a more homogeneous photon flux is achieved, resulting in shorter irradiation times, which in turn reduces side product formation. In addition, the large surface-area-to-volume (SA-V) ratio improves heat and mass transfer, allows easier handling of reactive chemicals, and permits automation, as well as inline analysis. Furthermore, the possibility to reuse the catalyst through immobilization, as well as the ease in product removal,^9^ makes reaction in flow, particularly attractive systems.^3,8,52^

While flow is widely used for photocatalysis, reports on photobiocatalysis in continuous systems are scarce. One example is the photodecarboxylation of palmitic acid catalyzed by a fatty-acid photodecarboxylase from *Chlorella variabilis* (*Cv*FAP) in a packed bed reactor setup.^53,54^ A higher productivity as compared with batch processes was reported in the continuous system. Another example is a dual-specie containing O_2_-producing *Synechocystis* with O_2_-respiring *Pseudomonas taiwanensis* VLB120 in a capillary biofilm reactor.^51^ Both strains heterologously expressed the CYP450 genes to catalyze the conversion of cyclohexane to cyclohexanol. The long duration (37 days) of film formation prior to the reaction and the low initial substrate concentration (1 mmol L^–1^) result in a rather low efficiency and space-time yield (STY = 0.2 g L^–1^ h^–1^), which represents a limitation of this otherwise very promising system. Our previous works using recombinant *Synechocystis* harboring monooxygenases^34,35,40^ were performed in batch reactors with sufficient aeration meaning enhanced oxygen concentration in the medium to drive the oxidation. However, since the reaction was performed in batch, limitations in the allowable cell density were encountered due to “self-shading”. Recently, we employed a coil reactor to reduce various substrates using recombinant cyanobacteria expressing the gene of the ene-reductase YqjM from *B. subtilis* to catalyze an NADPH-dependent and O_2_-independent reduction of various substrates.^49^ Compared with batch reactions, a higher specific activity (99.8 U g_DCW_^–1^) and STY (14.4 g L^–1^ h^–1^) were reported in flow.^49^ Furthermore, the high SA-V ratio (2000 m^2^ m^–3^) of the coil reactor would allow operations at increased cell densities as previously shown.^49^ Moreover, with its current design (*i.e.*, without additional air inlets), the rates at which photosynthetic oxygen could drive the oxidation could be determined.

In this work, we report the combination of flow chemistry and photosynthetic *in situ* oxygen generation to drive the C–H oxyfunctionalization of cyclohexanone **1a** to the polymer precursor ε-caprolactone **1b** catalyzed by a BVMO from *Burkholderia xenovorans* (BVMO_*Xeno*_). Our results show that *in situ* O_2_ production by *Synechocystis* directly into the medium can sustain the oxyfunctionalization reactions in systems where heterotrophic bacterial hosts fail due to the limited external oxygen supply. We aimed to determine the rates at which photosynthetic oxygen could solely drive the oxidation reaction. Hence, improvements in the design of the coil reactor to increase the oxygen concentration were not considered. Nevertheless, we believe that the *in situ* oxygen production can be easily combined with approaches facilitating an additional supply of external oxygen. The sustainability of the proposed reaction scheme was also detailed.

## 2 Experimental Section

### 2.1 Strains, Chemicals, and Cultivation Conditions

All chemicals were purchased from Sigma-Aldrich (Steinheim, Germany) or Carl-Roth GmbH (Karlsruhe, Germany) and used without further purification unless otherwise specified. Oxygen and temperature sensors were all purchased from Pyroscience GmbH (Aachen, Germany).

The *bvmo*_*Xeno*_ gene with an *N*-terminus 6x His-Tag was cloned to a pET22b plasmid and transformed to *E. coli* BL21 (DE3) as previously described.^35^ Overnight cultures (5 mL) were grown in Lysogeny Broth (LB medium) supplemented with ampicillin (100 μg mL^–1^) at 37 °C. The cells were then transferred to a terrific broth (TB) medium for protein production and grown at 25 °C and 120 rpm until they reached an optical density at 600 nm (OD_600_) of 0.6–0.8. Afterwards, 0.1 mM isopropyl β-d-1-thiogalactopyranoside (IPTG) was added to induce protein production and kept at 16 °C for 18 h.

*Synechocystis* cells harboring the same *bvmo*_*Xeno*_ gene but controlled by the light-inducible P_*cpc*_ promoter were cultivated under high light conditions.^35^ Briefly, *Synechocystis* cells were grown in Erlenmeyer flasks (working volume of 100 mL) in standard BG-11 supplemented with kanamycin (50 μg mL^–1^). A tunable LED lamp (CellDEG, Berlin, Germany) was used to illuminate the cultures at a light intensity of *ca.* 150 μmol of photons m^–2^ s^–1^. The flasks were placed on a rotary shaker at 140 rpm, and the cells were allowed to grow until an optical density at 750 nm (OD_750_) of *ca.* 1.0, which typically takes 3–4 days.

### 2.2 Coil Reactor Setup and Operation

The coil reactor setup previously described^49^ was modified for the oxyfunctionalization reaction. Figure S1 shows the actual coil reactor setup. Briefly, a food-grade polyvinyl chloride (PVC) tube (ID 2 mm, 1.5 m) having an internal volume of 4.7 mL was wrapped in a helical manner around a fluorescent lamp (OSRAM). The cell suspension (*V* = 10–15 mL) was maintained at an appropriate temperature using a thermostatic water bath. The temperature was monitored using a Pt100 Temperature Probe (TDIP15, Pyroscience). A peristaltic pump (Ismatec Masterflex) equipped with a Masterflex tube (No. 14) was utilized to deliver the cell suspension containing the substrate to the reactor. Standard Luer-lock connectors were used to connect the reactor with the cell/substrate suspension. The coil was held in place using 3D-printed tube holders (see Valotta et al.,^49^ for more information on the 3D printing of the holders). To monitor the net oxygen evolution during the reaction, oxygen sensors (Pyroscience) were placed in the reservoir, inlet, middle, and outlet of the coil reactor. For the reservoir, a robust oxygen probe (OXROB10) was used, while sterile oxygen flow-through cells (OXFLOW) connected to an optical fiber (SPFIB-BARE) were installed in the length of the reactor. The oxygen concentration throughout the coil reactor as well as the temperature inside the reservoir were monitored using an optical oxygen and temperature meter (FireSting-O_2_ 4 Channels, Pyroscience), respectively. All oxygen data were processed using the Pyroscience Workbench Software.

### 2.3 Whole-Cell Biotransformation

Biotransformations in *E. coli* harboring BVMO_*Xeno*_ were performed after overnight (18 h) protein production. Cells were centrifuged and concentrated to the required OD_600_ using a TB medium. The pH of the reaction mixture was adjusted to 7.5 using NaOH and the reactions were performed in the coil reactor (length= 1.5 m, internal diameter = 2 mm, internal volume = 4.7 mL) wrapped around a neutral white fluorescent lamp (OSRAM) as shown in Figure S1. The coil was placed 4 mm from the light source, delivering a light intensity of 300 μmol photons m^–2^ s^–1^ determined using a light meter (LI-250 A, LICOR Biosciences, Hamburg, Germany). The reservoir (total volume = 10–15 mL) containing the cell suspension was placed in a water bath maintained at 25 °C. Cells were delivered by a peristaltic pump set at a flow rate of 0.8 mL min^–1^. Before substrate addition (10 mmol L^–1^, substrate stock in ethanol), cells were allowed to run for at least 5 min in the coil reactor to monitor the dissolved oxygen concentration. After substrate addition, samples were taken periodically at the outlet, quenched in liquid nitrogen, and stored at −20 °C prior analysis.

Biocatalytic oxidation in *Synechocystis* was performed when the cultures reached an OD_750_≈ 1.0. The cell suspension was then concentrated by centrifugation (24 °C, 15 min, 3220*g*) and resuspended in fresh BG-11 to reach a desired OD_750_ and utilized directly for whole-cell biotransformations. The temperature of the water bath was set at 30 °C. The substrate (10 mmol L^–1^, substrate stock in ethyl acetate) was added after 5 min of cell suspension circulation in the coil reactor. Aliquots were similarly taken at different time points, quenched in liquid nitrogen, and stored at −20 °C prior analysis.

The batch reactions were performed by placing whole cells of either *E. coli* or *Synechocystis* in an Erlenmeyer flask or glass vial, respectively, and performing the reaction in a temperature-controlled incubator. In contrast to oxidations in the flow reactor, batch reactions were performed at a shaking speed of 130 rpm for *E. coli* and 160 rpm for *Synechocystis* to provide sufficient agitation and aeration of the medium.

### 2.4 Analytics

The concentration of all compounds was determined using gas chromatography equipped with a flame ionization detector (GC-FID, Shimadzu 2010) as previously described.^35^ Briefly, samples from whole-cell biotransformations were extracted with dichloromethane containing 2 mmol L^–1^ of *n*-decanol as an internal standard. After shaking for 1 min, the organic layer was dried with a spatula tip of anhydrous magnesium sulfate. The suspension was finally centrifuged and measured directly in the GC-FID equipped with a ZB-5 column. Separation was performed using a column oven program of 60 °C held for 5 min to 200 °C held for 3 min with a rate of 10 °C min^–1^ and finally to 300 °C held for 3 min at a rate of 300 °C min^–1^. Chlorophyll *a* content was determined using methanol extraction as described previously.^42^

### 2.5 Volumetric Mass Transfer Coefficient Determination

The volumetric mass transfer coefficient (*k*_L_*a*) of the coil reactor was determined in either TB or BG-11 buffer autoclaved prior to the measurements to remove any dissolved oxygen. Subsequently, the buffer was placed in the reaction vessel and further degassed by purging with N_2_ for at least 30 min. The reactor was then placed in the water bath set at 25 °C (for TB) or 30 °C (for BG-11) and was operated similarly to the coil reactions to mimic the reaction conditions. Light agitation was employed in the coil reactor by placing a stir bar (diameter 2 mm and length 12 mm) in the reservoir operated at 140 rpm similar to the biotransformation reaction. The oxygen concentration was also monitored by using the sensors (Pyroscience) attached to the reactor. The *k*_L_*a* was calculated using the dynamic response method^55^ with eqs 1 and 2.
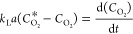
1
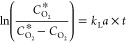
2where *k*_L_*a* is the volumetric mass transfer coefficient (h^–1^), *C*_O_2__^*^ is the saturated oxygen concentration (μmol L^–1^), *C*_O_2__ is the oxygen concentration (μmol L^–1^), and *t* is the time (h).

### 2.6 Space-Time-Yield Calculation

The space-time yield (STY) of the reaction systems was determined using eq 3, as previously described.^49^ For batch reactions, the STY was estimated from the volumetric productivity (*i.e.*, the amount of product formed after 1 h of reaction).
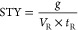
3where *g* is amount of product formed in one residence time (g), *V*_R_ is the internal reactor volume (L), and *t*_R_ is the residence time when operating in flow (h).

### 2.7 Atom Economy and E-Factor Calculations

The atom economy of the reaction was calculated by dividing the molecular weight of the product by the molecular weights of the reactants as previously described.^56^

The E-factor as well as the E^+^-factor (including CO_2_ emissions) were calculated using eq 4 as previously described.^31,57,58^
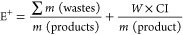
4where *m* denotes masses of wastes and products (kg), *W* is the electrical power used, and CI is the carbon intensity (kg_CO_2__ kW h^–1^).

## 3 Results and Discussion

### 3.1 Oxyfunctionalization in the Coil Reactor Using Recombinant BVMO in *E. coli*

To determine the extent to which *in situ* photosynthetic oxygen from *Synechocystis* expressing the BVMO_*Xeno*_ gene can drive an oxidation reaction without external oxygen supply, the BVMO_*Xeno*_ gene was first expressed in nonoxygen evolving, chemoheterotrophic *E. coli*. The BVMO reaction was performed in a coil reactor without supplementing oxygen, and the rates, as well as activities, were determined to compare with reactions mediated by *Synechocystis*.

**Figure 1:**
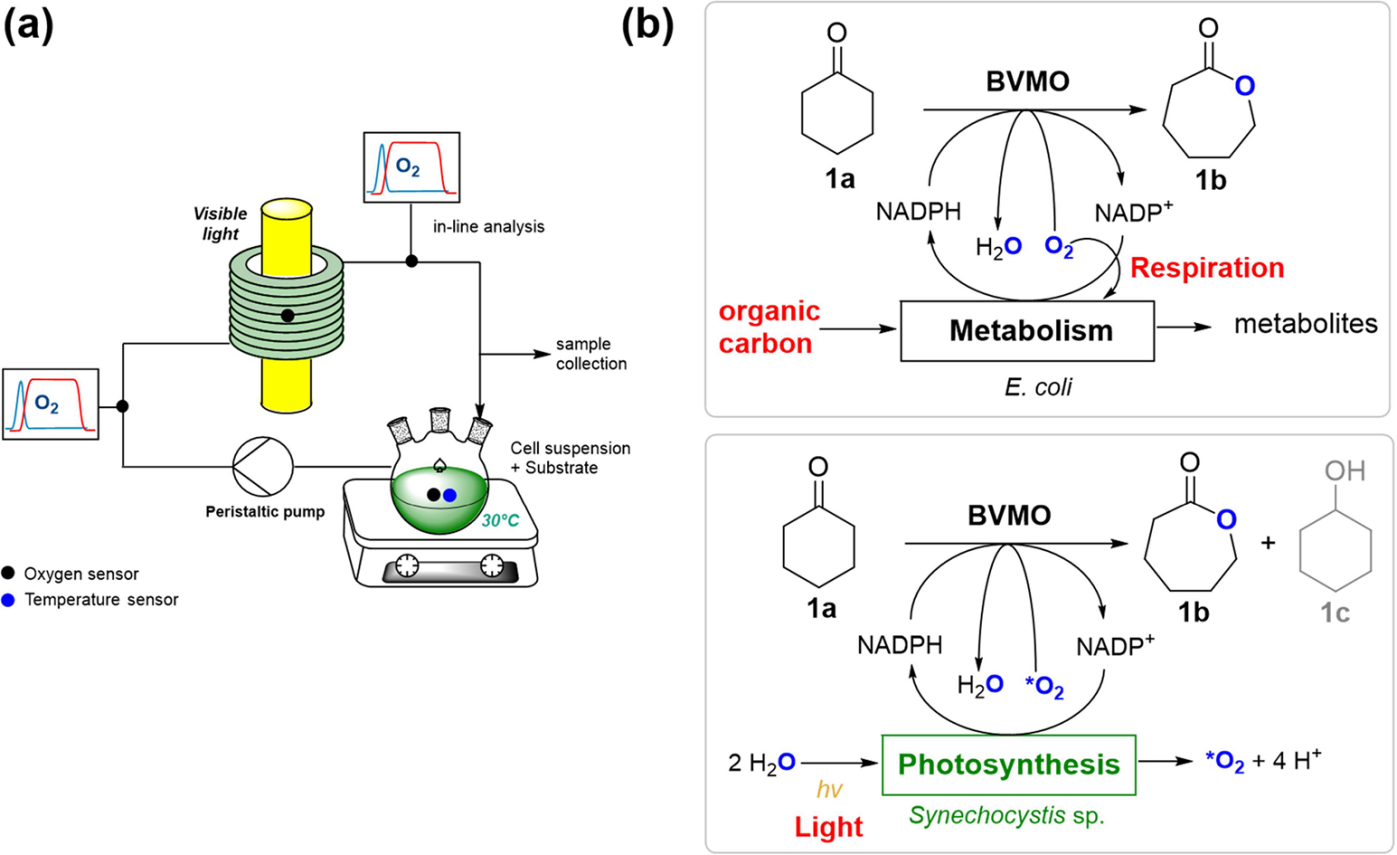
(a) Schematic diagram of the reaction setup and (b) comparison of the key aspects for sustainability (highlighted in red) for the oxyfunctionalization of **1a** in recombinant *E. coli* and *Synechocystis* harboring BVMOs. *O_2_ corresponds to *in situ* oxygen produced in the liquid medium by the cyanobacterium *Synechocystis* sp. through water splitting during photosynthesis which is readily available to drive oxidation reactions.

Figure 1a shows the coil reactor setup utilized in this study. This reactor was previously reported using transparent food-grade polyvinyl chloride tubing (inner diameter 2 mm) spirally wrapped around a light source.^49^ In this study, inline monitoring of the dissolved oxygen concentration in the solution at four positions of the coil, *i.e.*, reservoir, inlet, middle, and outlet (Figure S1 shows the actual coil reactor setup used), was set up and real-time dissolved oxygen was determined using the PyroScience Workbench Software. The reactor was operated at room temperature to reduce energy costs and to prevent overheating of the components. However, the reservoir containing the cell mixture and the substrate was submerged in a water bath set at an appropriate temperature (*i.e.*, 25 and 30 °C for recombinant *E. coli* and *Synechocystis*, respectively). Figure 1b shows the BVMO reaction mediated by either *Synechocystis* or *E. coli* harboring BVMO_*Xeno*_. In recombinant *E. coli*, organic carbon sources such as glucose or glycerol are added to regenerate NADPH and to serve as a sacrificial electron donor. On the other hand, using recombinant *Synechocystis*, NADPH is regenerated through photosynthesis and *in situ* O_2_ is generated through water splitting, released directly in the liquid medium, and subsequently utilized in the reaction.

Since oxidation catalyzed by BVMOs requires oxygen as a cosubstrate,^59,60^ we determined the oxygen transfer rate (OTR) in TB and BG-11 media, describing the rate at which O_2_ transfers between the gas and liquid phases for the coil reactor.^61^ Hence, estimation of the O_2_ volumetric transfer coefficient (*k*_L_*a*) was performed using the dynamic gassing-out method wherein the change of dissolved O_2_ concentration is monitored until saturation.^55^ The culture medium was degassed, and the dissolved oxygen in the reservoir was monitored until saturation using the same reaction conditions. Figure S2 shows a saturated oxygen concentration of *ca.* 225 μmol L^–1^ and a *k*_L_*a* of 2 h^–1^ both for TB and BG-11 media at 25 and 30 °C, respectively. Using the calculated *k*_L_*a*, a maximum OTR of 6.7 μmol min^–1^ L^–1^ (1 μmol min^–1^ = 1 U) was determined for the coil reactor (see SI for the calculation).

**Figure 2:**
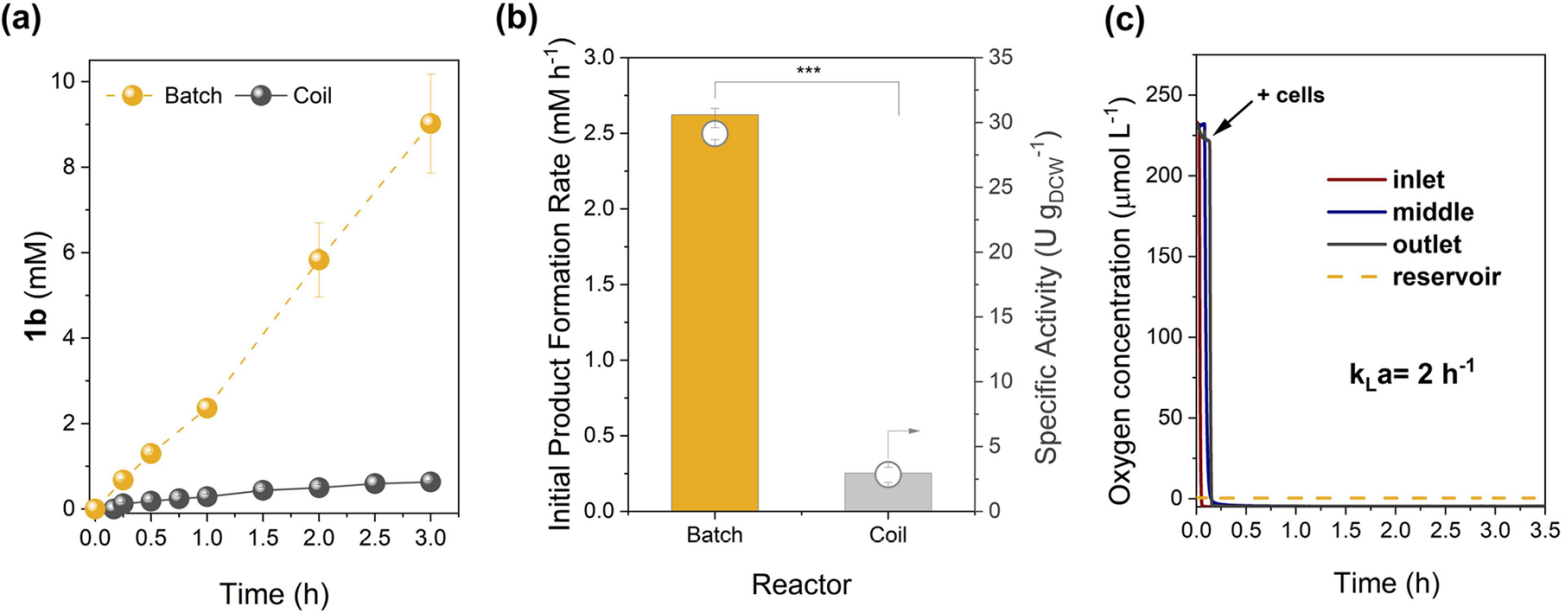
Whole-cell biotransformation of **1a** in the coil reactor mediated by BVMO_*Xeno*_ produced in *E. coli* BL21 (DE3). (a) Time profile of **1b** formation in batch and coil reactors; (b) initial product formation rates and specific activities normalized to cell dry weight in batch and coil reactors; and (c) dissolved oxygen concentration during oxidation measured at various points in the coil reactor. *Reaction conditions*: 25 °C, 0.8 mL min^–1^, 1.5 g_DCW_ L^–1^, 10 mmol L^–1^ initial concentration of **1a**. For batch reactions, the culture was placed in a shake-flask and agitated at 130 rpm to provide sufficient aeration; *N* = 2–3; *U* = 1 μmol min^–1^; product formation rates were calculated at <10% conversion. The *P* value was calculated using Welch’s *t* test (***P < 0.001).

The oxidation of **1a** (Figure 1b) by BVMOs using oxygen was used as the model reaction. In batch mode with sufficient aeration, *E. coli* BL21_BVMO_*Xeno*_ has a specific activity of 19 U g_DCW_^–1^ in the oxidation of **1a**.^35^ We then aimed to determine the rates in an oxygen-limited system by performing the whole-cell oxidation in the coil reactor using the optimum flow rate (0.8 mL min^–1^) determined previously in the coil reactor^49^ and the cell loading (1.5 g_DCW_ L^–1^) utilized in batch.^35^Figure 2a shows **1b** formation in flow using heterotrophic non-O_2_ evolving *E. coli*. Whole-cell bio-oxidation in the coil with recombinant *E. coli* harboring BVMO_*Xeno*_ showed minimal product formation (<1 mmol L^–1^ after 3 h, Figure 2a), which agrees with the low OTR of the reactor.

Using glycerol as an electron source for the biotransformation, we estimated a specific oxygen consumption rate of 119 U g_DCW_^–1^ (*i.e.*, 19 U g_DCW_^–1^ for oxygenation based on product formation at a cell density of 1.5 g_DCW_ L^–1^^35^ and 100 U g_DCW_^–1^ for endogenous respiration in growing cells).^29,30^ Considering the OTR, a maximum cell density of 0.056 g_DCW_ L^–1^ is allowable in the coil reactor. Since the biomass loading utilized during biotransformation was higher than this cell density, the oxygen demand of respiration and biotransformation exceeded the oxygen supply, resulting in low oxygen concentrations at the four measurement points (Figure 2c). Upon contact of the cell mixture with the sensor, the oxygen concentration dropped to zero from air-saturated oxygen level. For the cell density tested (1.5 g_DCW_ L^–1^), we calculated a theoretical maximal specific activity of 4.5 U g_DCW_^–1^, which is close to the one observed in the coil reactor (Figure 2b).

A parallel batch reaction was performed using the same cell density to compare the activity of *E. coli* in both reactors (Figure 2a,b). The reaction mixture in batch was agitated at 130 rpm to provide agitation and aeration.^62^Figure 2a shows 90% conversion of **1a** after 3 h comparable to what was previously observed using the same recombinant *E. coli*.^35^ Compared to the coil reactor, a 10-fold higher activity (29 U g_DCW_^–1^ in batch vs. 3 U g_DCW_^–1^ in coil, Figure 2b) was observed when the reaction was performed in batch with a much better oxygen supply. Thus, the observed higher activity of recombinant *E. coli* in batch compared to the coil is attributed to better aeration in the former, highlighting the significance of oxygen in BV oxidations.^60,63^ This was also confirmed by the drastic drop in the oxygen concentration in the coil reactor from air-saturated levels upon contact with the cell mixture (Figure 2c).

### 3.2 Oxygen from Photosynthetic Water-Splitting Drives Oxidation of **1a** in the Coil Reactor

Next, we investigated the reaction rate achievable by the substitution of the oxygen-consuming *E. coli* with oxygen-producing cyanobacteria, which sustain the reaction using homogeneous oxygen derived from photosynthetic water splitting. In addition to BVMO_*Xeno*_, the BVMO genes from *Parvibaculum lavamentivorans* (BVMO_*Parvi*_) and the well-studied cyclohexanone monooxygenase from *Acinetobacter* sp. (CHMO_*Acineto*_) were integrated into the genome of *Synechocystis* and expressed. The genes of BVMO_*Parvi*_ and BVMO_*Xeno*_ were expressed under the control of the strong light-inducible cpc promoter (P_*cpc*_). As P_*cpc*_ caused problems with the genetic stability of the *Synechocystis* strain bearing the CHMO gene (*data not shown*), the slightly weaker promoter psbA2 (P_*psbA2*_) was chosen.

**Figure 3:**
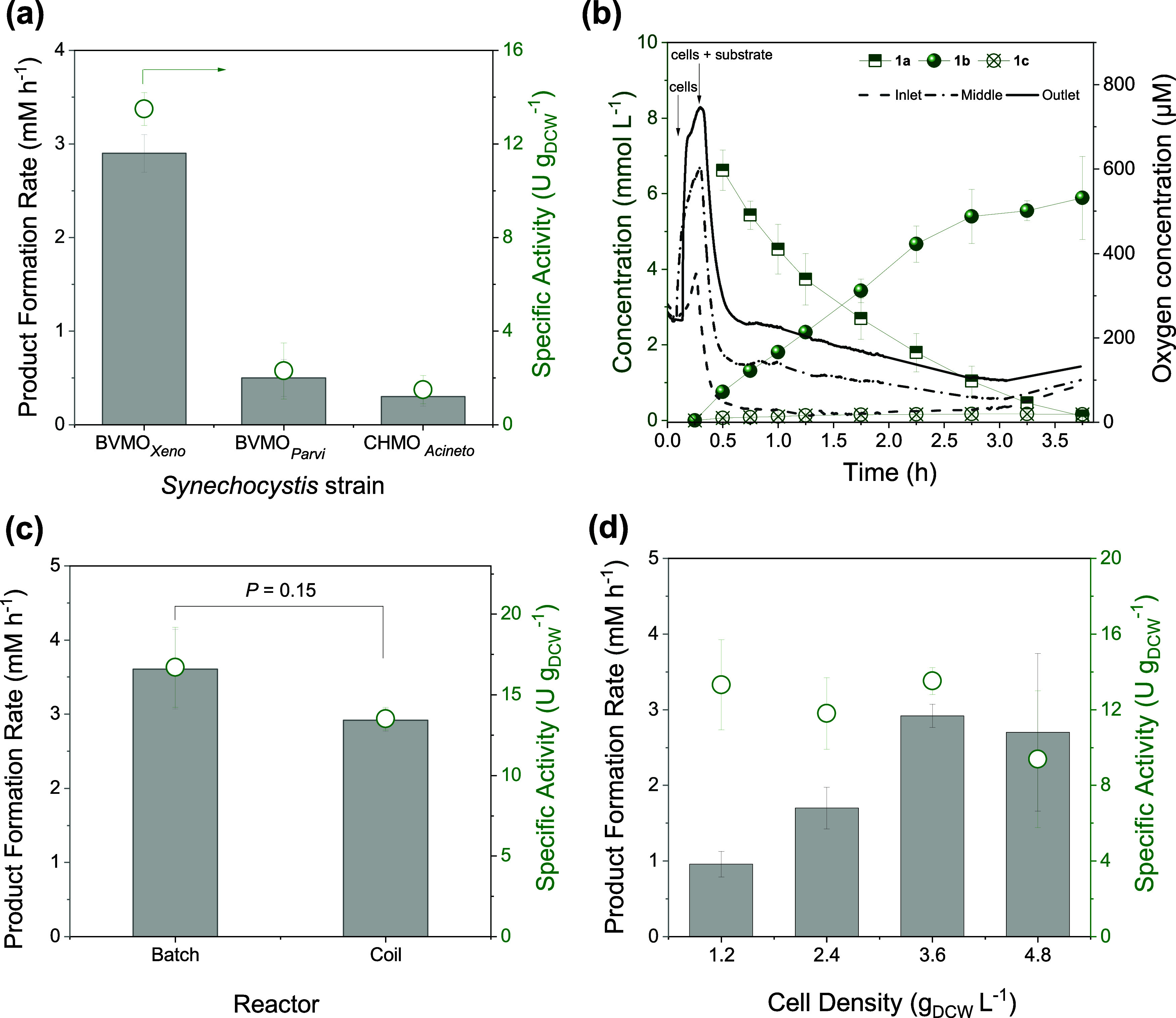
Whole cell biotransformation of **1a** in recombinant *Synechocystis* harboring BVMOs. (a) Comparison of activities using various BVMOs; (b) progress of the reaction mediated by Syn::P_*cpc*_BVMO_*Xeno*_ and oxygen evolution at several positions in the reactor; (c) comparison of activities and product formation rates between two reactor systems using Syn::P_*cpc*_BVMO_*Xeno*_ and (d) product rates and specific activities at various cell loading. *Reaction conditions*: 30 °C, 0.8 mL min^–1^, 3.6 g_DCW_ L^–1^, initial concentration of 10 mM **1a**, light intensity of 300 μmol photons m^–2^ s^–1^, *N* = 3 biological replicates. Batch reactions were performed at 160 rpm. *P* value was calculated using Welch’s *t* test.

Optimal operating conditions determined during ene-reduction^49^ using recombinant *Synechocystis* such as light intensity, flow rate, and cell density were also adapted for the oxidation reaction. Figure 3a shows the comparison of the product formation rates and specific activities in the whole-cell biotransformation of **1a** catalyzed by recombinant BVMOs in *Synechocystis*. The highest product formation rate was observed when the reaction was catalyzed by Syn::P_*cpc*_BVMO_*Xeno*_. High product formation rates in the oxidation of **1a** in batch have already been reported for BVMO_*Xeno*_.^35^ In the coil reactor without any external oxygen supply, a 6-fold higher specific activity was observed with BVMO_*Xeno*_ compared to other tested BVMOs. As expected, the oxygen concentration in the coil reactor did not decrease drastically, as was observed during biotransformations mediated by the heterotrophic whole-cell biocatalyst, *E. coli* (Figure 2c). In fact, an increase in oxygen concentration was observed in the outlet using Syn::P_*cpc*_BVMO_*Xeno*_ from air-saturated conditions (*ca.* 250 μmol L^–1^) (Figure 3b) when the cell mixture reached the sensor to a maximum of 420 μmol L^–1^. No significant differences in oxygen release were observed for all tested strains (Figure S3), indicating that the differences in activity did not stem from available oxygen supply but rather from the higher catalytic efficiency of BVMO_*Xeno*_. Moreover, the undesired production of cyclohexanol **1c** (which is also an inhibitor of Baeyer–Villiger monooxygenases) by endogeneous alcohol dehydrogenases in *Synechocystis*(34) was lower using BVMO_*Xeno*_ (Figure 3b). Possible reasons for the lower rate of the side reaction are the higher activity of Syn::P_*cpc*_BVMO_*Xeno*_ and the 10-fold lower *K*_M_ value of BVMO_*Xeno*_ towards **1a** compared to CHMO_*Acineto*_.^35^ For comparison, the biotransformation was similarly performed in batch mode, where the reaction vials were agitated at 160 rpm. In contrast to reactions with *E. coli* harboring BVMO_*Xeno*_, no significant difference in activities (*P* = 0.15) was observed between reactors when Syn::P_*cpc*_BVMO_*Xeno*_ was utilized to oxidize **1a** (Figure 3c). This shows that the overall oxygen supply was increased when photoautotrophic microorganisms were utilized in the reaction due to their ability to produce oxygen through water oxidation. Thus, in reactor systems without additional oxygen supplementation as in the coil reactor, utilizing cyanobacteria over O_2_-respiring heterotrophic *E. coli* is advantageous.

In addition, the amount of cells was optimized using the best-performing strain, Syn::P_*cpc*_BVMO_*Xeno*_. Figure 3b shows a representative time profile of the oxidation reaction at a cell density of 3.6 g_DCW_ L^–1^ (OD_750_ = 15). After substrate addition, the oxygen concentration decreased rapidly, indicating its consumption by the cells. This trend was observed in all the cell densities tested (Figure S4). The initial rate of product formation increased concurrently upon increase in cell concentration reaching a maximum of 3 mmol L^–1^ h^–1^ at a cell density of 3.6 g_DCW_ L^–1^ (Figure 3d). Further increase in the cell loading did not show improvement in the specific activity which could be attributed to self-shading effects.^42,49^ This cell density was also deemed optimal in the reduction of 2-methylmaleimide using *Synechocystis* harboring YqjM from *B. subtilis* carried out in a coil reactor.^49^ By performing the reaction in flow with a coil reactor having an internal diameter of 2 mm, uniform light distribution and mixing were achieved, and a higher cell density (delivering higher product formation rates) can be utilized as compared to batch reactions without significant decrease in activity.

### 3.3 Oxygen Production is Higher than Photoreduction in *Synechocystis*

**Figure 4:**
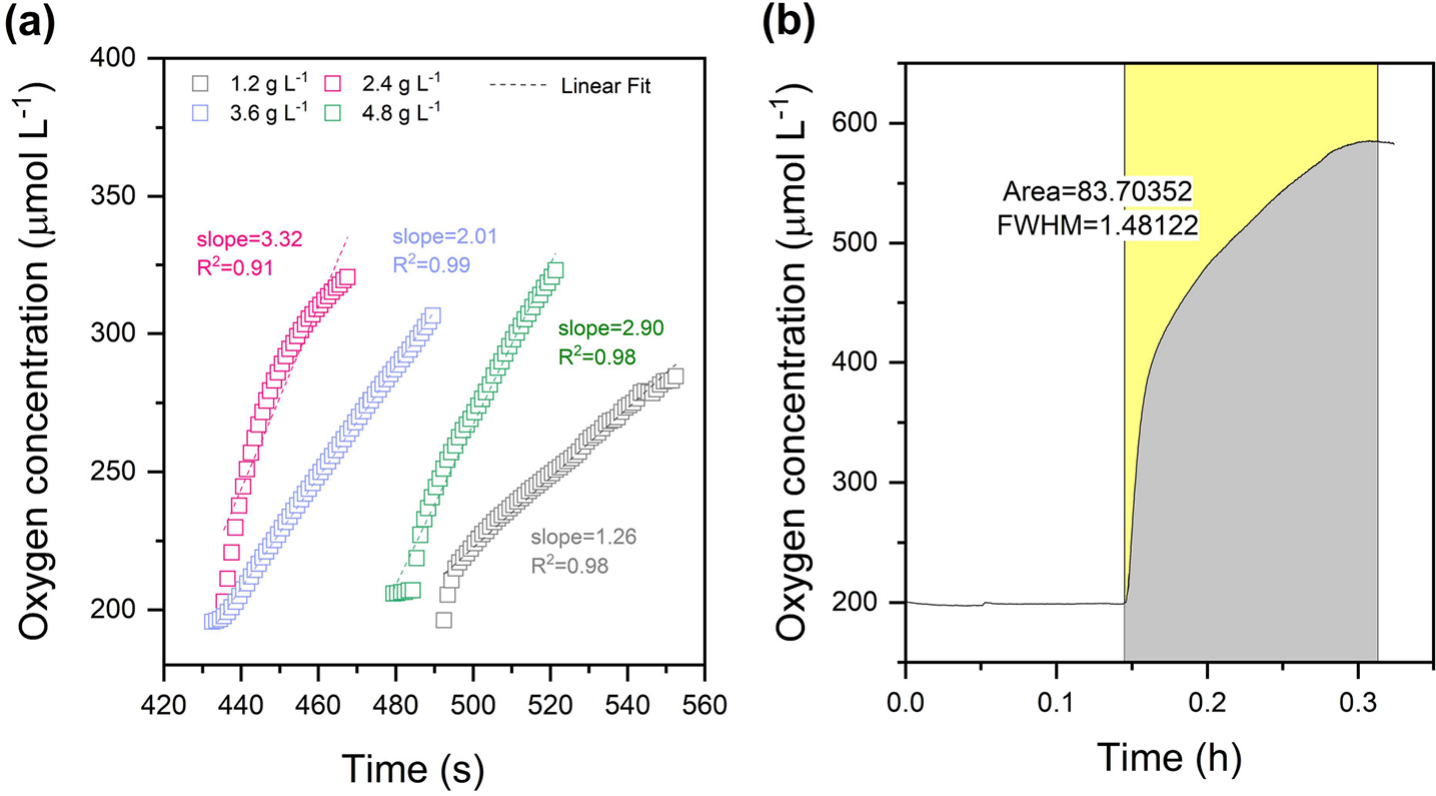
(a) Initial rates of oxygen production determined at the outlet after approximately one cycle by calculating the slope at each cell densities and (b) cumulative oxygen production at the outlet using Syn::P_*cpc*_BVMO_*Xeno*_ at a cell density of 3.6 g_DCW_ L^–1^ before substrate addition. The area under the curve was integrated using OriginPro software and corrected by subtracting the area below air-saturated oxygen levels.

We have shown that whole-cell biotransformations using Syn::P_*cpc*_BVMO_*Xeno*_ in the continuous system (Figures 3b and S4) showed a positive net oxygen evolution, which is a striking difference compared to reactions with *E. coli* using glycerol as an electron source (Figure 2). Hence, we calculated the initial rates of net oxygen increase at each cell density from the oxygen production at the outlet of the coil reactor. It should be noted that the cells were not acclimated to darkness prior to the measurement, and the monitoring was performed at the same time as the oxidation reactions. Figure 4 shows the slopes calculated for each cell density and a representative curve for cumulative oxygen release. Table 1 shows the initial rates of net oxygen evolution in the coil using Syn::P_*cpc*_BVMO_*Xeno*_ at different cell densities during the reaction prior to substrate addition.

**Table 1:** Rates of Net Oxygen Evolution in the Coil Reactor at Different Cell Densities during Oxidation of **1a** Mediated by Syn::P_*cpc*_BVMO_*Xeno*_

cell density (g_DCW_ L^–1^)	rate of net oxygen evolution^a^ (mmol L^–1^ h^–1^)	normalized to chl*a*^b^ (μmol h^–1^ mg_chl*a*_^–1^)	cumulative O_2_ production^c^ (μmol L^–1^ h)
1.2	4.6 ± 0.1	51.0 ± 2.7	48.7 ± 5.2
2.4	10.1 ± 1.2	46.4 ± 8.9	43.2 ± 5.5
3.6	15.4 ± 1.8	65.0 ± 1.2	74.7 ± 3.5
4.8	13.0 ± 4.8	15.5 ± 4.0	63.8 ± 8.5

a Initial rates of net oxygen evolution were calculated at the onset of oxygen increase up to 1 min at each cell density in Figure S4.

b Chlorophyll *a* content in each cell density was determined as a percentage of cells in the coil reactor over the reservoir volume (4.7 mL over 15 mL in the reservoir). Net oxygen evolution was considered at the outlet of the reactor.

c Cumulative oxygen evolution was calculated as the area under the curve during the onset of oxygen increase until the addition of **1a**.

The oxygen evolution increased concurrently with the increase in cell density until OD_750_ = 15 (3.6 g_DCW_ L^–1^). Furthermore, an increase in cell density did not improve the oxygen concentration, which could be attributed to self-shading similarly shown in Figure 3d. A positive net oxygen evolution was observed for all cell loadings tested with a maximum of 65 ± 1.2 μmol O_2_ h^–1^ mg chl*a*^–1^ at 3.6 g_DCW_ L^–1^. Similar to *E. coli*, (photo)respiratory pathways are present in photosynthetic microorganisms such as cyanobacteria and microalgae reducing the photosynthetically derived O_2_ to other compounds.^64^ From Table 1, a positive net oxygen evolution was shown. Therefore, we note that oxidation reactions mediated by recombinant *Synechocystis* harboring BVMOs carried out in the coil reactor are not considered O_2_-limited, resulting in higher production rates compared to recombinant *E. coli* under oxygen-limiting conditions.

*In situ* oxygen production from water splitting in Photosystem II (PSII) is dependent on light intensity.^65^ We have shown that *Synechocystis* harboring BVMOs showed a maximum positive net oxygen evolution of 65 μmol O_2_ mg chl*a*^–1^ h^–1^ (Table 1). This is comparable with net oxygen evolution rates (54–90 μmol O_2_ mg chl*a*^–1^ h^–1^) determined in WT *Synechocystis* at the light intensity range of 150–300 μmol photons m^–2^ s^–1^.^65^ In contrast, growing chemoheterotrophic cells such as *E. coli* have a respiration rate of *ca.* 100 U g_DCW_^–1^ restricting the applicable biomass concentration to 15 g_DCW_ L^–1^.^29,30^ Under these conditions, all available O_2_ is consumed for respiration resulting in a drastically reduced productivity which was also shown in this study (Figure 2). By utilizing photoautotrophic cyanobacteria, the maximum achievable productivity was greatly increased, expanding the net oxygen accumulation rate to include oxygen evolution rate (OER) from water oxidation.^30^

### 3.4 Improving the Space-Time-Yield of the Enzymatic Oxidation using the Flow Mesoreactor

Light availability is a crucial factor in cyanobacterial growth and, consequently, in photobiocatalytic biotransformations. Cyanobacterial light utilization is severely hampered at higher cell densities due to light reflection, dissipation, and shading effects^66,67^ requiring reactions to be performed at cell densities below 2 g L^–1^ which in turn results in low STY. Scaling-up photobiocatalytic reactions (involving reactor diameters in the centimeter range) remains challenging due to the drastic decrease in light transmittance.^52^ The performance of the coil reactor was then compared with a batch reactor at similar cell loading and with various reactor geometries utilizing photoautotrophic microorganisms for selected oxidation reactions, particularly in large reactors (Table 2). Additionally, studies using heterotrophic *E. coli* cells for oxidation were also listed to compare the oxidation rates in non-O_2_ evolving hosts.

**Table 2:** Comparison of Enzymatic Oxidation Reactions in Batch and Continuous Reactors

item	specie	enzyme	substrate	reactor	*V* (cm^3^)	air supply (L min^–1^)	SA-V^a^ (m^2^ m^–3^)	sp. act. (U g_DCW_^–1^)	STY^b^ (g L^–1^ h^–1^)	ref
1	*Synechocystis*	BVMO	cyclohexanone	batch	1		345	16.7 ± 2.5	0.4 ± 0.1	this study
2		BVMO	cyclohexanone	coil (Flow)	4.7		2000	13.5 ± 0.7	2.8 ± 0.4	this study
3		BVMO/Lactonase	cyclohexanone	batch	2000	2^c^	9^d^	10.3^e^	6.4 × 10–2^e^	(36)
4		BVMO	cyclohexanone	batch	2000	2	9^d^	30.0 ± 0.3	0.2 ± 0.1	(37)
5		CYP450	cyclohexane	capillary film (flow)	1.2		1333	n.a.	0.2	(51)
6		AlkBGT	nonanoic acid methyl ester	batch	1		250	0.9 ± 0.1	n.a.	(33)
7		AlkBGT	nonanoic acid methyl ester	batch	3000	1.8	9^d^	5.6 ± 0.1	1.7 × 10^–5^^e^	(69)
8	*E. coli*	CHMO	cyclohexanone	batch	3000	2	250	18	0.8	(24)
9		CHMO	bicyclo[3.2.0]hept-2-en-6-one	batch	1500	50	n.a.	41^e^	1.9	(25)
10		CHMO	bicyclo[3.2.0]hept-2-en-6-one	fed-batch	1500	1.5	n.a.	n.a.	0.9^e^	(27)
11		CHMO	bicyclo[3.2.0]hept-2-en-6-one	batch (bubble reactor)	1000	3^e^	n.a.	96	1	(63)
12		CHMO	bicyclo[3.2.0]hept-2-en-6-one	batch (double sintered sparger)	50,000	115^e^	n.a.	82^e^	1	(26)
13		CPMO	4-methylcyclohexanone	batch	1000	1	n.a.	n.a.	1	(70)
14	cell-free	tyrosinase	tyrosol	flow	10	2.2 × 10^–4^	n.a.	n.a.	0.04^e^	(68)
15		GOX	glucose	flow	20	1–10 × 10^–3^	1200	23 g product g enzyme^–1^	182	(28)

a SA-V pertains to surface area to volume ratio of the reactor utilized.

b For batch reactions, the STY considered pertains to volumetric productivity.

c Additional CO_2_ supply of 20 mL min^–1^.

d Estimated from the dimensions of a Labfors 5 photobioreactor (www.infors-ht.com).

e Calculated from the reference; n.a.–not available; CYP450–cytochrome oxidase P450; CPMO–cyclopentanone monooxygenase; GOX–glucose oxidase.

A higher STY (7-fold) was observed when oxidation was performed in the coil reactor compared with batch using recombinant *Synechocystis* expressing BVMO_*Xeno*_ (Table 2, Items 1 and 2). This could be attributed to a 6-fold higher SA-V ratio of the coil reactor compared to the batch reactor (2000 vs 345 m^2^ m^–3^) permitting efficient light distribution. Moreover, the light diffusion path is significantly reduced thereby improving mixing and enhancing mass transfer by utilizing the mesoscale reactor.^49^ To the best of our knowledge, this is the highest STY reported in oxidative biotransformations mediated by recombinant *Synechocystis*. It compares favorably to the rates and volumetric productivities obtained by others (Table 2, Items 3 and 5). This was also observed in the hydroxylation of tyrosol performed in a coil reactor showing a 2.9-fold higher product yield as compared to the batch reactor (Item 14).^68^ Oxidation reactions in larger reactors (*e.g.*, continuous stirred tank reactors, fermenters) (Items 3 and 4) require a supply of O_2_ even when utilizing recombinant *Synechocystis*, and STYs were considerably lower as compared to reactions in flow.^36,37,39^ On the other hand, the necessity to supply oxygen in oxidation reactions mediated by recombinant *E. coli* contributes to mass transfer limitations restricting the product yields, STY, and catalyst turnovers prompting operations with O_2_-enriched air^60^ or pressurized reactors.^28^ This would entail constructing bioreactors with complex and expensive equipment to regulate the pressure.^13^ The simple design of the coil reactor operated at ambient conditions (T and P) described in this study coupled with *in situ* O_2_ production from recombinant *Synechocystis* sets it apart from other flow reactors. Increasing the throughput in the coil reactor could also be carried out by running reactors in parallel *(i.e.*, numbering up) without altering the amount of absorbed light.^52^ For industrial applications, we envision that oxygen-producing whole-cell photobiocatalysis in flow will be combined with oxygen-permeable column materials and elevated pressure, thus providing an optimal gas supply for oxygen-dependent enzymatic reactions.

*Synechocystis* has been successfully employed for whole-cell biotransformations involving heterologous oxidoreductases. In particular, BVMOs produced in cyanobacteria have shown specific activities ranging from 2 to 60 U g_DCW_^–1^ through heterologous expression^34,37^ and photosynthetic electron transport chain manipulation.^35^ The aforementioned reactions were all performed in batch, resulting in low STY. In this work, we attempted to improve the STY by employing a continuous reactor with an increased surface-to-volume ratio to enhance mass transfer. In terms of energy efficiency and resources, the reaction system was nonsterile and the PVC tubing was compatible with the cells, hence sterilization by autoclaving was not required. The whole-cell system utilizing recombinant BVMO in *Synechocystis* can sustain 3 mmol L^–1^ h^–1^ of product formation using *in situ* O_2_ in an active oxidation reaction. This reaction system can potentially be coupled with other oxygen-dependent reactions without requiring an additional oxygen supply (*e.g.*, glucose oxidase).

### 3.5 Evaluating the Sustainability of the Proposed Continuous Oxidation in Recombinant *Synechocystis*

The sustainability of the proposed continuous oxidation using recombinant BVMOs in *Synechocystis* was determined using the Zero Pass CHEM21 metrics toolkit.^71^ In addition, the E-factor was also calculated according to the principles of green chemistry.^58^ The reaction system was compared to the heterotrophic-mediated oxyfunctionalization of **1a** using recombinant *E. coli*. In Table 3, the advantages of using recombinant *Synechocystis* to drive the oxidation in O_2_-limited systems compared with heterotrophic *E. coli* can be seen. Using water as a sacrificial electron donor instead of glycerol significantly increased the atom efficiency of photosynthetic O_2_-driven oxidation of **1a** mediated by recombinant *Synechocystis*. We calculated an atom economy of 98 and 60% for *Synechocystis*- and *E. coli*-mediated BVMO reactions, respectively. The higher atom efficiency of cyanobacteria-mediated reactions was also reported for the hydroxylation of testosterone compared with *E. coli*.^40^ Admittedly, the atom economy of biotransformation in a heterotrophic bacterium is challenging to calculate, as it is not known which part of the electrons will be dedicated to NADPH generation and biomass formation, respectively. Nevertheless, typical biotransformation reactions use a stoichiometric or even a molar excess of the carbon source. Moreover, compared with the chemical production of **1b** using hazardous and expensive organic peracids,^21^ photobiocatalytic **1b** formation mediated by recombinant *Synechocystis* uses only water under mild conditions and renewable resources. Furthermore, BVMO-mediated oxidation produces only water as a byproduct.

**Table 3:** Zero Pass Green Metric for the Biocatalytic Oxidation of **1a** to the Polymer Precursor **1b** in the Coil Reactor without an External Oxygen Supply

strain	yield^a^ (%)	conversion (%)	selectivity (%)	atom economy (%)	reaction mass efficiency (%)	solvents	health & safety
*Syn*_BVMO_*Xeno*_	79.1	92.6	85.4	98.2	77.2	aqueous buffer, substrate stock solution in ethyl acetate	no red flags
*E.coli*_BVMO_*Xeno*_	9.5	27.4	35.5	60.0	5.5	aqueous buffer, substrate stock solution in ethanol	no red flags

a Using the actual substrate concentration determined by GC-FID.

For E-factor calculation, raw materials such as the buffer ingredients and the cells were included (see Table S1). A simple E-factor value of 10.7 was calculated for the reaction mediated by recombinant *Synechocystis*. On the other hand, a high E-factor of 753.6 was determined for the BVMO reaction using recombinant *E. coli* due to the low product formation in the continuous coil reactor. The BVMO reaction was also compared with the state-of-the-art reaction producing a kilogram scale of lactones from *rac-*bicyclo[3.2.0]hept-2-en-6-one using a bubble column reactor.^26^ This reaction was mediated by heterotrophic *E. coli* harboring CHMO_*Acineto*_. Oxygen and glycerol were supplemented to provide the oxidant and regenerate the cofactors, respectively. Due to this, an atom economy of 53.5% was calculated similar to that obtained using *E. coli*_BVMO_*Xeno*_. A simple E-factor of 12.2 was calculated for the reaction (Table S1) at the same reaction duration of 3 h, which is slightly higher than the simple E-factor calculated from the BVMO reaction mediated by *Synechocystis*. Hence, our proposed system could be potentially more sustainable. However, these numbers are only estimations and several parameters should be considered when switching to pilot scale. Specifically, the inclusion of contaminated water (containing even low concentrations of chemicals and microorganisms)^31^ in the calculation as shown in Table S1, significantly increased the E-factor with water accounting for *ca.* 90% of the contribution. To decrease the E-factor, further work should include increasing the product amount by either fed-batch addition of the substrate or *in situ* product removal. Another route to decrease the E-factor is to use immobilized whole cells packed in columns and reuse the cells. The E^+^ factor was also evaluated considering the energy demands such as pumping, stirring, and heating.^57^ Considering 242 g CO_2_ produced per kWh,^72^ an E^+^ factor of 63 × 10^3^ was calculated accounting for majority of the wastes generated. The significant contribution of electricity-related CO_2_ emissions was also observed in the preparation and purification of unspecific peroxygenases (UPO) from *Agrocybe aegerita* (r*Aae*UPO) recombinantly produced in *Pichia pastoris*.^57^ To alleviate the high E^+^ factor in cyanobacterial biotransformations, efforts should be aimed at efficient illumination systems and reduced reaction times. The latter could be improved by increasing the catalytic efficiency of the enzyme or using cyanobacterial strains with improved oxygen production.

## 4 Conclusions

Whole-cell bio-oxidations mediated by heterotrophic microorganisms either growing or resting cells suffer from oxygen limitation due to endogenous respiration limiting the reaction rates. This is a severe limitation for the implementation of O_2_-dependent enzyme reactions in flow systems. This study demonstrates that homogeneous oxygen directly supplied into the reaction medium by the photosynthetic cyanobacterium, *Synechocystis* harboring a BVMO efficiently supports an O_2_-dependent whole-cell biotransformation reaction in a continuous reactor system.

By performing the photo(bio)oxidation in a coil reactor having an improved surface-area-to-volume ratio (increased photon flux density), cell densities up to 4.8 g_DCW_ L^–1^ could be utilized without drastic decrease in the activity. The aforementioned cell density is 2-fold higher compared with the optimum cell densities utilized in batch with *Synechocystis* harboring CHMO_*Acineto*_ highlighting the advantages of conducting photoredox catalysis in a coil reactor. Furthermore, this work demonstrates that whole-cell recombinant *Synechocystis* harboring BVMOs can provide a homogeneous oxygen supply at a maximum rate of 15.4 mmol L^–1^ h^–1^ (65 μmol h^–1^ mg chl*a*^–1^) together with reducing equivalents to fuel an oxidation reaction without relying on external oxygen supply in a continuous photobioreactor. A product formation rate of 3 mmol L^–1^ h^–1^ was attained without supplying additional oxygen for the sustainable production of an important platform chemical. This is especially relevant in upscaling reaction systems using light and higher cell densities to achieve high STY. In this work, a 7-fold increase in STY was achieved by performing the reaction in flow compared to batch. The sustainability of the reaction was also improved showing an atom economy of 98% by using only water as the electron donor compared with *E. coli* where the necessary molar excess of carbon source significantly reduces this sustainability parameter. Finally, this work highlights the advantages of cyanobacteria as a noteworthy host for the heterologous production of enzymes aimed at oxygen- and NADPH-dependent reactions.

## Supporting Information Available

Media for cultivation and whole-cell biotransformation, illuminated coil reactor setup, determination of the *k*_L_*a* of the coil reactor, oxygen concentration determination in the coil reactor and calculation of the rate of oxygen per cell density tested, calculation of the E-factor (PDF)

## Abbreviations

*E. coli*: **Escherichia coli**
E-factor: environmental factor
*Synechocystis*: *Synechocystis* sp. PCC 6803
BVMO: Baeyer–Villiger monooxygenases
CYP450: cytochrome P450 monooxygenases
NADPH: β-nicotinamide adenine dinucleotide phosphate
*Cv*FAP: fatty acid decarboxylase from *Chlorella variabilis*
STY: space-time-yield
BVMO_*Xeno*_: Baeyer–Villiger monooxygenase from *Burkholderia xenovorans*
OTR: oxygen transfer rate
*k*_L_*a*: volumetric transfer coefficient of O_2_
DCW: dry cell weight
BVMO_*Parvi*_: Baeyer–Villiger monooxygenase from *Parvibaculum lavamentivorans*
CHMO_*Acineto*_: cyclohexanone monooxygenase from *Acinetobacter* sp.
P_*cpc*_: light-inducible cpc promoter
P_*psbA2*_: light-inducible psbA2 promoter
PSII: photosystem II
OER: oxygen evolution rate
SA-V: surface-area-to-volume
r*Aae*UPO: unspecific peroxygenase from *Agrocybe aegerita*
