# Supplementary material for: Light-Driven Photobiocatalytic Oxyfunctionalization in a Continuous Reactor System without External Oxygen Supply
Source: ACS Sustain Chem Eng. 2025 Mar 5;13(10):3939–50. doi: 10.1021/acssuschemeng.4c08560 (PMC11921032; doi:10.1021/acssuschemeng.4c08560)
Supplement: Supplementary file 1 — sc4c08560_si_001.pdf [file sc4c08560_si_001.pdf]

ELECTRONIC SUPPORTING INFORMATION

Light-driven Photobiocatalytic Oxyfunctionalization in a Continuous Reactor System without External Oxygen Supply

Lenny Malihan-Yap,[†] Qian Liang,[†] Alessia Valotta,[‡] Véronique Alphand[#], Heidrun Gruber-Woelfler,[‡] Robert Kourist[†, ǂ,*]

[†]Institute of Molecular Biotechnology, Graz University of Technology, Petersgasse 14, 8010 Graz, Austria. E-mail: kourist@tugraz.at.

[‡]Institute of Process and Particle Engineering, Graz University of Technology, Inffeldgasse 13, 8010 Graz, Austria.

[#]Aix Marseille Univ, CNRS, Cent Marseille, iSm2, F-13397 Marseille, France.

[ǂ]ACIB GmbH, Krenngasse 37, 8010 Graz, Austria

Supporting Information Contents:

Total number of pages: 9 (including cover page)

Total number of Figures: 4

Total number of Tables: 1

Table of Contents

I. Media for Cultivation and Whole-Cell Biotransformations S3

II. Illuminated Coil Reactor Set-up S4

III. Determination of the Oxygen Transfer Rate in the Coil Reactor S5

IV. Photosynthetic Oxygen Production using Recombinant Cyanobacteria harbouring various BVMOs S6

V. Oxygen Release using *Synechocystis* sp. harbouring $BVMO_{Xeno}$ during whole-cell biotransformation at various cell loadings S7

VI. Determination of oxygen transfer rate using the measured volumetric transfer coefficient S8

VII. Calculation of E-value S8

References S9

I. Media for Cultivation and Whole-Cell Biotransformations

1. For *E. coli*:

For overnight cultures, Lysogeny Broth (LB) Medium containing 10g L^{-1} Tryptone, 5 g L^{-1} Yeast Extract, 5 g L^{-1} NaCl was utilized. For protein production, Terrific Broth (TB) Medium containing 12 g L^{-1} Peptone, 24 g L^{-1} Yeast Extract, 4 mL L^{-1} Glycerol, 2.2 g L^{-1} KH_2PO_4, 9.4 g L^{-1} K_2HPO_4 was used. Solid media from LB were also prepared containing 15 g L^{-1} agar supplemented with 100 µg mL^{-1} ampicillin.

2. For *Synechocystis* sp.

For growth and whole-cell biotransformations, standard BG-11 medium[1] was used. Solid media were also prepared using BG-11 containing 0.16 g L^{-1} $Na_2S_2O_3$. *Synechocystis* sp. strains containing the expression cassette for BVMO are supplemented with 50 µg mL^{-1} kanamycin as selective antibiotic.

II. Illuminated Coil Reactor Set-up

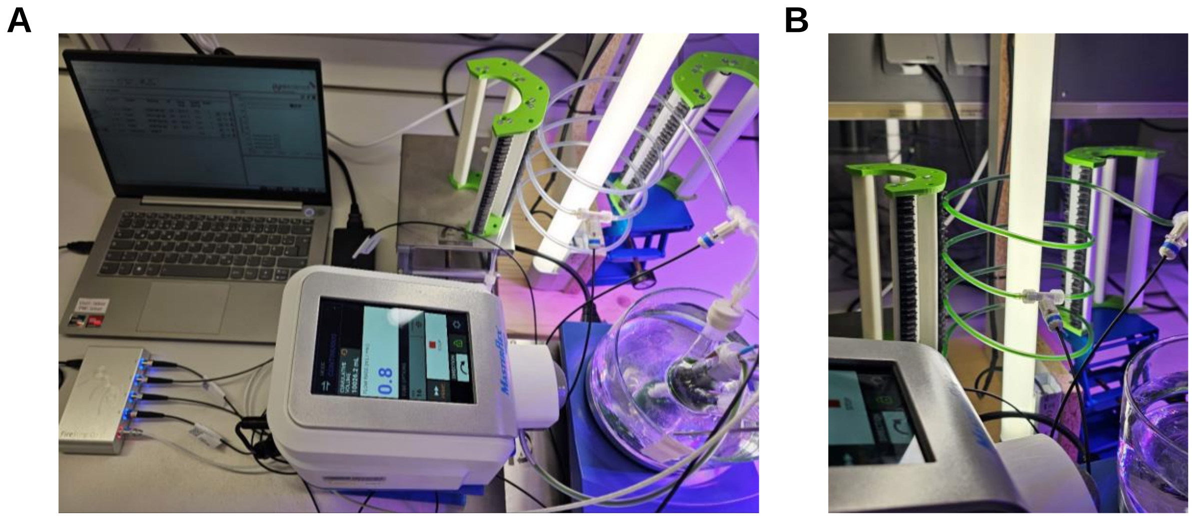

Fig. S1. A) Actual experimental set-up of the coil reactor with attached oxygen and temperature sensors and (b) Coil reactor held using 3D-printed holders and illuminated using a fluorescent lamp. The cell and substrate solution were delivered to the reactor using a peristaltic pump set at a specified flowrate. The reservoir is placed in a water bath set at an appropriate temperature.

III. Determination of the Oxygen Transfer Rate in the Coil Reactor

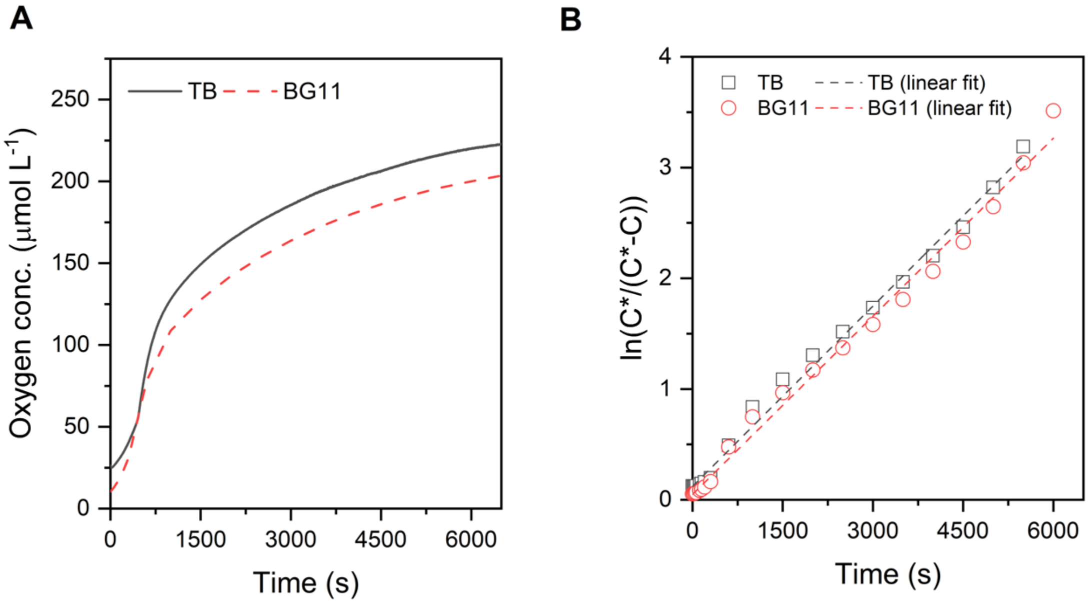

Fig. S2. Determination of oxygen transfer rate (k_La) in the coil reactor. (A) Time profile of dissolved oxygen concentration in the medium operated in the coil reactor and (B) Linear regression plot used for the calculation. *Reaction conditions:* 15 mL reservoir volume (25 mL three neck flask), 140 rpm using a stir bar (length of 12 mm and thickness of 2 mm).

IV. Photosynthetic Oxygen Production using Recombinant Cyanobacteria harbouring various BVMOs

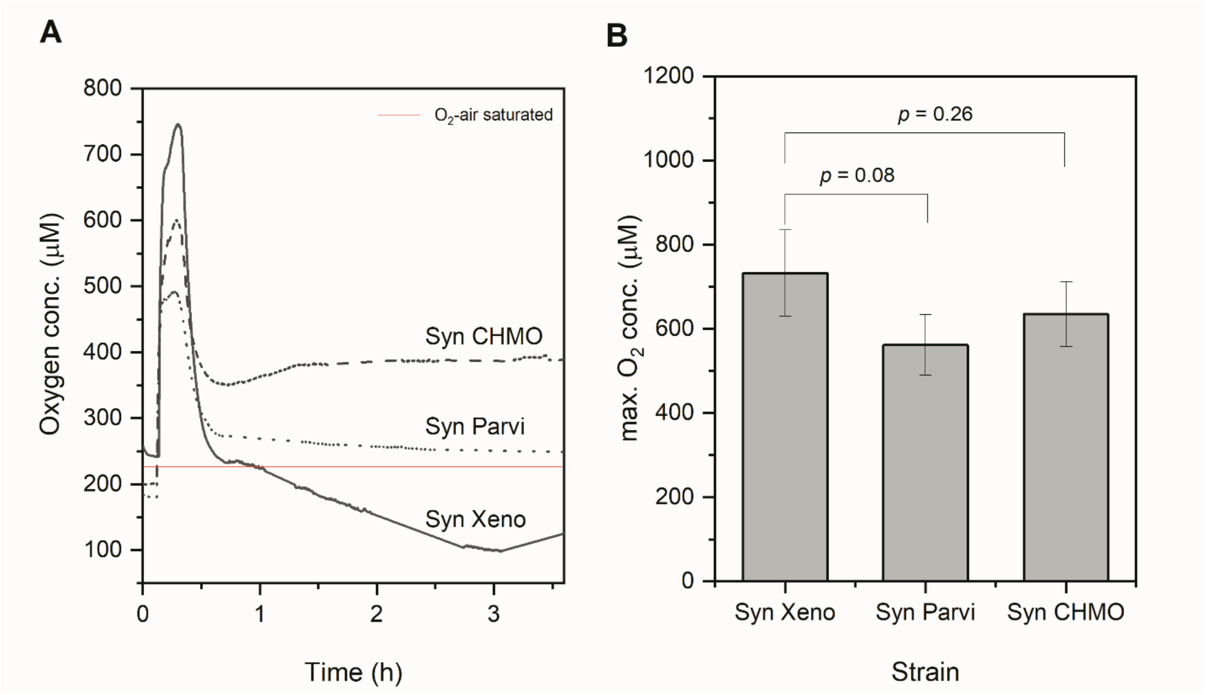

Fig. S3. (A) Oxygen release during whole-cell biotransformation of **1a** mediated by various BVMOs in *Synechocystis* measured at the outlet of the coil reactor; (B) Comparison of maximum oxygen concentration at the reactor for all tested BVMOs. *P* values were calculated using Welch's *t*-test and correspond to maximum oxygen release; *N* = 3 biological replicates.

V. Oxygen Release using *Synechocystis* sp. harbouring BVMO$_{Xeno}$ during whole-cell biotransformation at various cell loadings

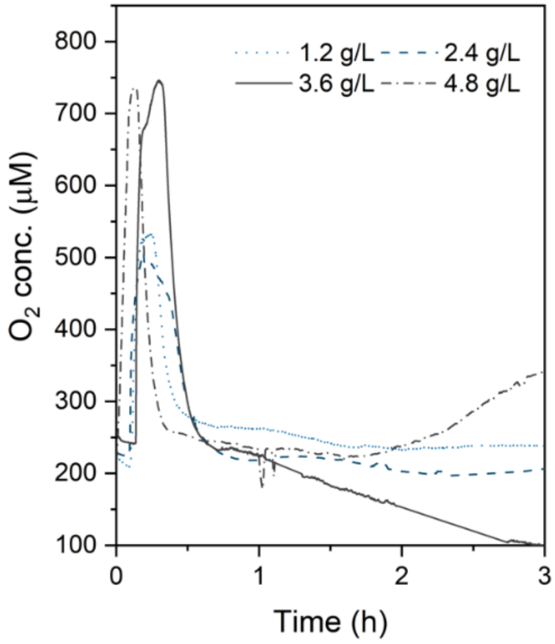

Fig. S4. Time profile of oxygen release at different cell loadings using Syn::P$_{cpc}$BVMO$_{Xeno}$ in the whole-cell biotransformation of **1a** carried out in the coil reactor. *Reaction conditions:* 30 °C, 0.8 mL min^{-1}, 300 µmol photons m^{-2} s^{-1}, V_{int}= 4.7 mL, initial concentration of 10 mM **1a**; *N*= 3.

VI. Determination of oxygen transfer rate using the measured volumetric transfer coefficient

The oxygen transfer rate was determined using equation (1) where k_La was determined via dynamic gassing out method and the concentrations of oxygen, *i.e.* saturated and minimum were monitored using the oxygen sensors.

$$OTR = k_L a \left(C^*_{O2} - C_{O2}\right)$$

$$OTR = 2\ h^{-1}(225\text{-}26)\frac{\mu mol}{L} = 0.4\ \frac{mmol}{L\text{-}h} = 6.7\ \frac{\mu mol}{L\text{-}min}$$

From the OTR, the maximum allowable cell density was calculated from the specific oxygen consumption rate of growing recombinant *E. coli* harbouring $BVMO_{Xeno}$. An oxygen consumption rate of 119 U g_{DCW}^{-1} was estimated to account for respiration of growing cells (100 U g_{DCW}^{-1})[2,3] and the oxygenase activity (19 U g_{DCW}^{-1})[4].

$$6.7\ \frac{U}{L} \times \left(\frac{g}{119\ U}\right) = 0.056\ {g_{DCW}}/{L}$$

VII. Calculation of E-value

For the calculation of the E-factor, only the oxidation reaction was considered but not the cell cultivation. The carbon dioxide emissions for electricity used for the calculation of the E^+ factor was based on 242 g CO_2 per kWh for 2023 in EU.[5]

Table S1. Estimation of the E-factor for the bio-oxidation of 1a mediated by either recombinant Synechocystis or *E. coli* performed in the coil reactor.

**Parameter**	***Synechocystis***	***E. coli***	
	This study	This study	Hilker et al. [6]
Reaction volume, mL	15	15	50,000
Operating time, h	3	3	3
Medium ingredients, g	0.043	0.790	2,955
Water, g	14.900	14.190	44,898
Cells, g_{DCW}	0.054	0.023	350
**1a** fed, g	0.015	0.015	900[a]
**1b** formed, g	0.010	0.001	360[a]
O_2 supplied, g	-	-	537[b]
E-factor (without water)	10.71	753.58	12.17
E-factor (with water)	1,579.14	13,701.77	136.89

[a]substrate is *rac*-bicyclo[3.2.0]hept-2-en-6-one; [b]O_2 is supplied using a sintered-metal aeration sparger

References

(1) Büchsenschütz, H. C.; Vidimce-Risteski, V.; Eggbauer, B.; Schmidt, S.; Winkler, C. K.; Schrittwieser, J. H.; Kroutil, W.; Kourist, R. Stereoselective Biotransformations of Cyclic Imines in Recombinant Cells of *Synechocystis* Sp. PCC 6803. *ChemCatChem* **2020**, *12* (3), 726–730. DOI:10.1002/cctc.201901592.

(2) Duetz, W. A.; van Beilen, J. B.; Witholt, B. Duetz_Proteins in Their Natural Environment-Potential and Limitations of Microbial Whole-Cell Hydroxylations in App Biocat. *Curr Opin Biotechnol* **2001**, *12*, 419–425.

(3) Hoschek, A.; Schmid, A.; Bühler, B. *In Situ* O_2 Generation for Biocatalytic Oxyfunctionalization Reactions. *ChemCatChem* **2018**, *10* (23), 5366–5371. DOI:10.1002/cctc.201801262.

(4) Erdem, E.; Malihan-Yap, L.; Assil-Companioni, L.; Grimm, H.; Barone, G. D.; Serveau-Avesque, C.; Amouric, A.; Duquesne, K.; De Berardinis, V.; Allahverdiyeva, Y.; Alphand, V.; Kourist, R. Photobiocatalytic Oxyfunctionalization with High Reaction Rate Using a Baeyer-Villiger Monooxygenase from *Burkholderia xenovorans* in Metabolically Engineered Cyanobacteria. *ACS Catal* **2022**, *12* (1). DOI:10.1021/acscatal.1c04555.

(5) Brown, S.; Jones, D. *European Electricity Review 2024*.

(6) Hilker, I.; Wohlgemuth, R.; Alphand, V.; Furstoss, R. Microbial Transformations 59: First Kilogram Scale Asymmetric Microbial Baeyer-Villiger Oxidation with Optimized Productivity Using a Resin-Based in Situ SFPR Strategy. *Biotechnol Bioeng* **2005**, *92* (6), 702–710. DOI:10.1002/bit.20636.
